# Knowledge, Attitude, and Practice towards Chelating Agents in Endodontic Treatment among Dental Practitioners

**DOI:** 10.3390/dj11070156

**Published:** 2023-06-21

**Authors:** Anna Mikheikina, Nina Novozhilova, Maria Polyakova, Inna Sokhova, Anastasia Mun, Alexandr Zaytsev, Ksenia Babina, Irina Makeeva

**Affiliations:** 1 Department of Therapeutic Dentistry, I.M. Sechenov First Moscow State Medical University (Sechenov University), 119991 Moscow, Russia; novozhilova_n_e@staff.sechenov.ru (N.N.); polyakova_m_a_1@staff.sechenov.ru (M.P.); sokhova_i_a@staff.sechenov.ru (I.S.); mun.nastya@mail.ru (A.M.); babina_k_s@staff.sechenov.ru (K.B.); makeeva_i_m@staff.sechenov.ru (I.M.); 2 Institute of Linguistics and Intercultural Communication, I.M. Sechenov First Moscow State Medical University (Sechenov University), 119991 Moscow, Russia; zaytsev_a_b@staff.sechenov.ru

**Keywords:** chelating agents, endodontics, smear layer, survey, root canal irrigants

## Abstract

The use of chelating agents (CAs) in the endodontic irrigation protocol is required to dissolve the inorganic components of the smear layer. We aimed to assess the knowledge, attitude, and practice of dental professionals regarding the use of CAs during root canal treatment. A cross-sectional anonymous online survey was conducted among specialized endodontists and general dentists who routinely perform endodontic treatment and work in government-funded or private clinics in Moscow. The 8 min survey consisted of four parts: basic demographic data, knowledge (five items), attitude (four items), and practice (five items). We collected 376 completed questionnaires; a majority of the respondents were general dentists (87.5%) and worked in private clinics (77.4%). Most respondents (83.5%) showed a fair knowledge of the CAs used in endodontics, while 16.5% showed a poor knowledge of the topic. Small yet significant differences were found between endodontists and general practitioners and between dentists employed by private and government-funded clinics. A majority of dental practitioners (83%) demonstrated a positive attitude towards the use of CAs in endodontic treatment, and there were no differences among the study subgroups. Almost a third of the respondents always used chelating solutions during endodontic treatment, while 17% of the respondents did not use them at all. There were significant differences in this parameter between dentists working in private and government-funded clinics. Practice significantly correlated with attitude towards chelating agents and with knowledge of the topic. In conclusion, dental practitioners demonstrated a fair knowledge of CAs. Despite a positive attitude, 71% of the respondents did not use CAs for all endodontic patients.

## 1. Introduction

The primary goal of root canal treatment (RCT) is the prevention and/or elimination of periapical infection [1]. This can be achieved by mechanical debridement, shaping, irrigation, and filling of the entire root canal system.

Proper irrigation of the root canals during shaping and cleansing is one of the key factors in endodontic success. It is aimed at removing debris and microorganisms as well as the smear layer. The smear layer is formed on the root canal walls during endodontic instrumentation [2]. It is composed of dentin debris and pulpal remnants and microorganisms, i.e., organic and inorganic components. In the literature, there are two positions regarding the need to remove the smear layer during root canal treatment. On the one hand, the smear layer may contribute to bacterial adhesion and colonization and result in root canal obturation failure [3,4,5]. Therefore, smear layer removal may increase the penetration of irrigants and medicaments into the complex anatomy of the root canal system and promote good adaptation of the obturating material to the root canal wall [6,7]. On the other hand, its removal increases dentinal permeability, allowing microorganisms to penetrate into the dentinal tubules, compromising treatment outcome [8,9]. Despite this controversy, the literature in general sides toward smear layer removal before obturation [10,11]. Therefore, an ideal endodontic irrigant solution should not only disinfect dentin and dentinal tubules, but also dissolve pulp tissue and dentin debris in order to remove the smear layer [12]. However, none of the existing irrigating solutions can be regarded as optimal.

Surveys conducted in different countries have shown that sodium hypochlorite is the most widely used root canal irrigant due to its activity against endodontic pathogens and its pulp-dissolving properties [13,14,15,16,17,18]. However, sodium hypochlorite dissolves only organic tissue, and thus the supplementary use of demineralizing or chelating agents (CAs) is required to dissolve the inorganic components of the smear layer [19,20]. The mechanism of action of chelating agents is based on their reaction with calcium ions and the formation of soluble calcium chelates, thus resulting in the decalcification of dentin [21]. Five to ten milliliters [22,23,24] of 10–17% solution of ethylenediaminetetraacetic acid (EDTA) disodium salt applied for at least 1 min [20,25,26] is most commonly recommended for this purpose [13,27,28,29,30,31,32,33,34,35]. Different techniques may be used to enhance the action of EDTA, e.g., ultrasonic [7,36] or sonic activation [37], negative pressure irrigation [38], laser activation [39], and manual dynamic agitation [40]. At the same time, EDTA activation may also cause the surface disintegration of root canal dentin [41].

Although EDTA does not exhibit a significant antimicrobial effect [42,43,44], it disrupts the biofilm polysaccharide matrix [45,46], thus enhancing the anti-biofilm effect of sodium hypochlorite. At the same time, combined irrigation with these solutions results in a chemical reaction, leading to the loss of the antimicrobial and tissue-dissolving properties of sodium hypochlorite [27,47,48]. Moreover, the use of EDTA can cause dentin erosion and the softening and denaturation of collagen fibers, thus decreasing the adhesion of the obturating material to the root canal walls [49,50,51].

Apart from EDTA, other chelating agents may be used in endodontics, e.g., citric acid [52,53,54] and maleic acid [55,56]. Both acids have been reported to effectively remove the smear layer; however, maleic acid provided better removal of the smear layer from the apical third [57] and demonstrated antibiofilm potential [43,58,59]. Like EDTA, both citric and maleic acids react with sodium hypochlorite and decrease its effect [47,60]. Recently, weak chelators such as etidronic acid and tetrasodium EDTA have been proposed for root canal irrigation to overcome this limitation [19,47,61]. Weak chelators can be mixed with sodium hypochlorite without loss of its antimicrobial and tissue-dissolving properties [47,61,62,63,64]. A mixture of sodium hypochlorite with one of these CAs can be potentially used as the only irrigant throughout root canal chemomechanical instrumentation [47,61,62,65,66]. However, further studies are needed to establish its clinical effect.

To sum up, although extensive research has been conducted on the use of CAs in root canal treatment, there is no single accepted evidence-based protocol of root canal irrigation describing the choice of irrigants and their sequence. Moreover, the surveys on endodontic irrigation among dental professionals have shown that some clinicians are still not aware of the importance of smear layer removal [13,67,68]. To the best of our knowledge, there have been no earlier knowledge, attitude, and practice (KAP) surveys among dental practitioners directly focusing on CAs.

The aim of our study was to assess the knowledge, attitude, and practice of dental professionals regarding the use of chelating agents during root canal treatment.

## 2. Materials and Methods

A cross-sectional online survey was conducted between 20 October 2022 and 10 January 2023 after ethical approval (2202) was obtained from the Ethics Committee of Sechenov University, Moscow, Russia. A questionnaire was developed in Russian based on the contemporary literature on the topic [19,31,48,69,70]. It was further reviewed for face and content validity by 4 independent reviewers who were members of the dental faculty. A pilot test of the questionnaire was initially performed on a group of 30 dental professionals who were excluded from the main sample. Feedback was collected regarding the clarity and order of questions and the time taken to complete the questionnaire. Modifications were made according to the comments received to enable better understanding.

The 8 min survey consisted of four parts: basic demographic data, knowledge, attitude, and practice regarding the use of CAs in endodontics.

The demographic data included questions on gender, years of clinical experience, specialty (endodontist or general practitioner), and workplace (government-funded or private clinic).

The knowledge section consisted of 5 questions on the types of CAs used in endodontics and EDTA concentrations and properties. Two items were single-choice questions, and three items were multiple-choice questions. To obtain a total knowledge score, each correct answer was given a score of 1 and each incorrect answer was given a score of 0. The scores of all items were summed, with a maximum total score of 24. The total knowledge score was categorized as poor if it was lower than 50% (<12 points), fair if it was between 50% and 75% (≥12 and ≤17 points), and good if it was higher than 75% (>17 points).

The attitude section consisted of 4 statements concerning the use of CAs in endodontics. The study participants could agree or disagree with the statements using a five-point Likert scale. Two items were positive-attitude statements (ranging from 5—“strongly agree”—to 1—“strongly disagree”), and two items were negative-attitude statements (ranging from 1—“strongly agree”—to 5—“strongly disagree”). The overall attitude was regarded as positive if the score was higher than 50% (>10 points) or negative if the score was lower than 50% (≤10 points).

The practice section consisted of 5 questions on the actual use of CAs by the respondents while performing endodontic procedures, with a maximum possible score of 8.

Convenience sampling was used to recruit the participants through personal contacts and social media platforms. Data collection was performed using Google forms. A link was sent to dental professionals using different methods, including emails, text messages, and posts on professional social networks. All data were collected anonymously and treated confidentially. The respondents were informed that completion of the questionnaire indicated consent to participate in the study and to have their responses (used in aggregate form) published in a journal article.

The targeted samples included specialized endodontists and general dentists who routinely performed endodontic treatment and worked in government-funded and private clinics in Moscow, Russia.

The total number of the study population was estimated to be around 5000 basing on the information from sberhealth.ru.

A minimum sample size of 357 respondents was calculated assuming a 95% CI and a margin of error of 5% using the following formula:

$$\mathrm{Sample}\;\mathrm{size}=\frac{\frac{z^{2}\times p\left(1-p\right)}{e^{2}}}{1+\left(\frac{z^{2}\times p\left(1-p\right)}{e^{2}N}\right)},$$

where

*z* (z-score) = 1.96 (95% CI);
*p* (standard deviation) = 0.5;
*e* (a margin of error) = 0.05;
*N* (a population size) = 5000.

Data manipulation was performed through MS Excel version 16.71 (23031200) and R version 3.6.0 (26 April 2019) with the following packages: “doBy,” “rstatix,” “stats,” and using RStudio version 2023.03.0 + 386 (2023.03.0 + 386). The data were presented as means, medians, standard deviations, 25th and 75th percentiles (quantitative variables), and counts and percentages (qualitative variables). A Shapiro–Wilk test was used to assess the normality of distribution of the quantitative variables (knowledge, attitude, and practice overall scores), and a Mann–Whitney U test was performed to compare these variables in the groups. Fisher’s exact test was used to compare the proportions. Spearman’s rank correlation coefficient was calculated to reveal pair-wise correlation between knowledge, attitude, and practice scores.

## 3. Results

Of the 378 questionnaires received, 2 were omitted because they were incomplete. A majority of the participants were female, accounting for 69.4% (*n*  =  261) of the study sample, while men accounted for 30.6% (*n*  =  115) of the study sample. Almost half of the participants (42.6%) had less than 5 years of clinical experience, 97 (25.8%) had 5–10 years of clinical experience, 75 (19.9%) had 11–20 years, and 44 (11.7%) had more than 20 years of clinical experience. A majority of the respondents were general dentists (*n*  =  329, 87.5%) and worked in private clinics (*n* = 291, 77.4%). Table 1 shows a detailed distribution of the participants’ demographic characteristics.

Endodontists demonstrated better knowledge compared with general practitioners, as specified in Table 2. The median number of correct answers was 14 (12; 15) out of the possible 24, with small yet significant differences between endodontists and general practitioners (*p* = 0.01185) and between dentists employed in private and government-funded clinics (*p* = 0.01742). A majority of the respondents (83.5%) showed fair knowledge of the use of CAs in endodontics, while 16.5% showed poor knowledge of the topic.

Table 3 shows the distribution of responses to knowledge questions about chelating solutions across the study subgroups. Most respondents in all subgroups correctly identified EDTA properties and concentrations. However, a significantly smaller proportion of general practitioners (76.4%) compared with endodontists (93.6) correctly chose the concentrations of EDTA solutions used for root canal irrigation (*p* = 0.004399). None of the respondents chose all the chelating solutions used in endodontics correctly, and only 9% of the respondents chose five of the six solutions correctly. A small proportion of the dentists were aware of the effects of the interaction between EDTA and sodium hypochlorite (23.1%) and between EDTA and chlorhexidine (5.3%).

**Figure 1:**
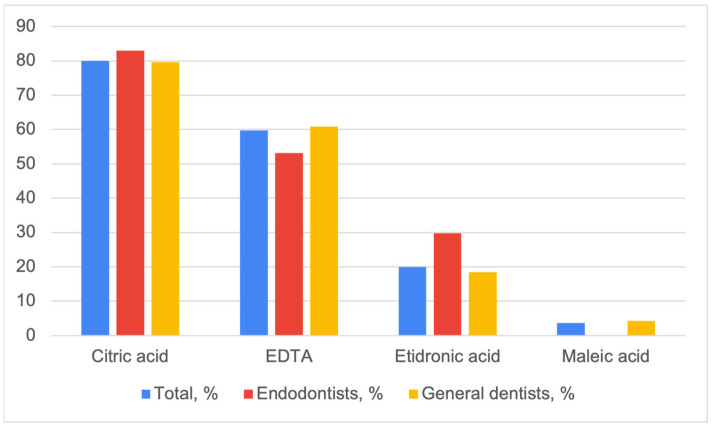
Respondents’ knowledge of acids used as chelating agents in endodontics (EDTA—ethylenediaminetetraacetic acid; GDs—general dentists).

Out of the four correct options, the most recognized CAs used in endodontics were citric acid and EDTA (Figure 1). Almost 20% of the respondents were aware that etidronic acid could be used as an endodontic CA. However, only 14 respondents (3.7%) were aware of the use of maleic acid for that purpose.

A majority of dental practitioners (83%) demonstrated a positive attitude towards the use of CAs in endodontic treatment (Table 4). No differences were found between the mean attitude scores of endodontists and general dentists (*p* = 0.5555) or between those of clinicians working and private and government-funded clinics (*p* = 0.4837).

More than half of the respondents agreed or strongly agreed that root canal irrigation with sodium hypochlorite should be alternated with root canal irrigation using CAs (Table 5). At the same time, 27% of the respondents considered CA use appropriate only in sclerotized root canals, approximately 45% of dentists believed that CAs can decrease the antimicrobial action and tissue dissolution capacity of sodium hypochlorite, and 37% of dentists thought that CAs adversely affect the mechanical properties of dentin.

Table 6 shows the use of chelating substances by practitioners during RCT. No differences were found in mean practice scores between the study subgroups.

**Figure 2:**
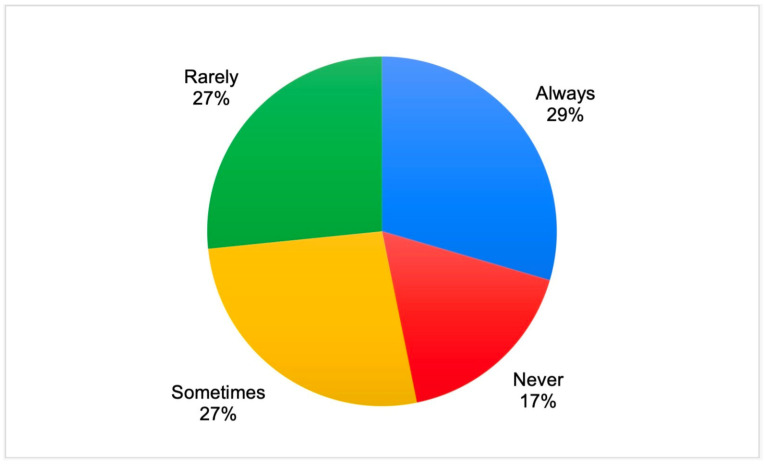
The frequency of chelating solutions used by the survey participants.

Almost a third of the respondents always used chelating solutions during endodontic treatment, while 17% of the respondents did not use chelating solutions at all (Figure 2). There were no significant differences in this parameter between the study subgroups. However, a greater proportion of the dentists working in private clinics indicated that they used chelating irrigating solutions “always” (30.6%) or “sometimes” (29.9%), while a greater proportion of the dentists working in government-funded clinics used chelating solutions “rarely” (34.1%) or “never” (24.7%).

**Figure 3:**
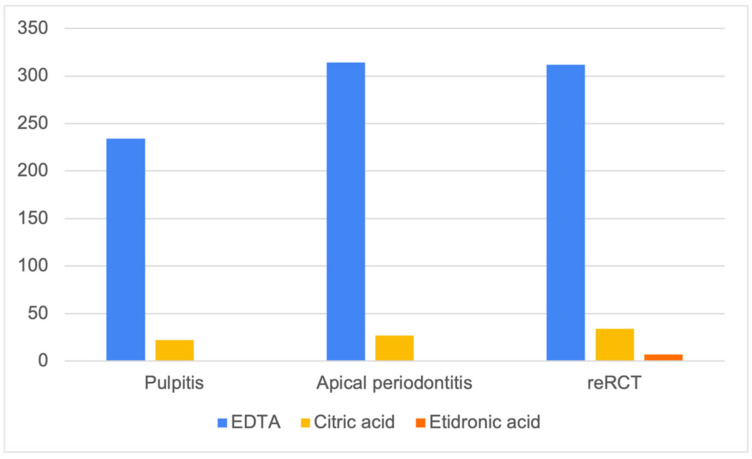
The use of chelating solutions in different clinical cases (EDTA—ethylenediaminetetraacetic acid; reRCT—root canal retreatment).

EDTA was the most popular chelating solution used for root canal irrigation in RCT and root canal retreatment (reRCT) (Figure 3). A small proportion of dentists reported the use of citric acid in both RCT and reRCT. Interestingly, seven dentists (1.9%) also reported the use of etidronic acid in reRCT.

**Figure 4:**
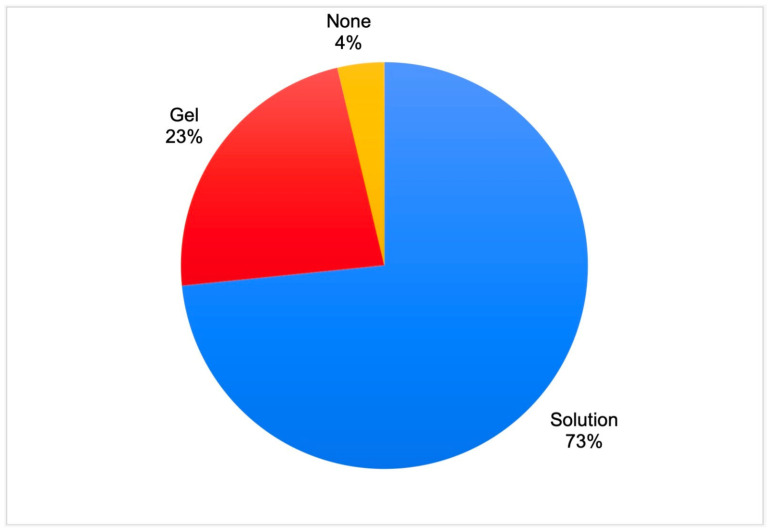
The forms of ethylenediaminetetraacetic acid preferred by the respondents.

Regarding the preferred EDTA forms, a majority of the respondents used EDTA solution, 23% used EDTA gel, and 4% did not use this CA (Figure 4). Dental practitioners working in private clinics preferred liquid EDTA forms (81%), while dentists working in government-funded clinics used liquid (47.1%) and gel-type (43.5%) forms in equal proportions (*p* < 0.001). Endodontists used EDTA solution more frequently (87.2%) than general dentists (71.4), although the differences were not significant (*p* = 0.06661).

**Figure 5:**
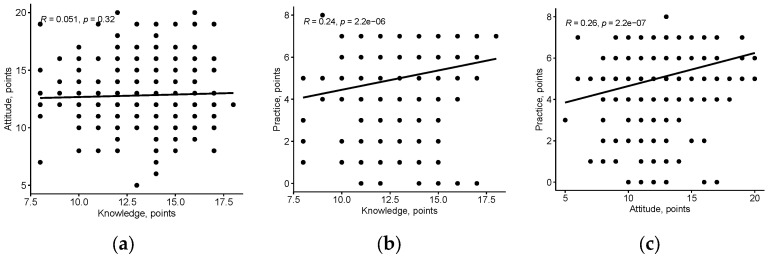
The correlation between knowledge, attitude, and practice of dental practitioners towards the use of chelating agents in endodontics: (**a**) the correlation between attitude and knowledge; (**b**) the correlation between practice and knowledge; (**c**) the correlation between practice and attitude.

There was a significant weak positive correlation between attitude and practice (r = 0.26, *p* < 0.001) and between knowledge and practice (r = 0.24, *p* < 0.001) (Figure 5). However, no correlation was found between knowledge and attitude (r = 0.051, *p* < 0.32).

## 4. Discussion

The aim of our study was to assess the knowledge, attitude, and practice of Moscow-based dental professionals towards the use of CAs during root canal treatment. We surveyed endodontists and general dentists employed in private and government-funded clinics. Surprisingly, none of the respondents in our study had a good knowledge of the topic. Dental practitioners had a fair knowledge of chelating endodontic agents, which was significantly better among specialized endodontists than among general dentists. No difference was found between the levels of knowledge of dentists employed in private and government-funded clinics.

To the best of our knowledge, there have not been any cross-sectional KAP surveys on CAs among dental practitioners that are directly comparable to ours. Earlier surveys partially comparable to ours focused mainly on preferred endodontic and irrigation practices. Most of these studies also reported a fair knowledge of the endodontic standards [71,72,73,74]. Bansal et al. reported that 43% of endodontists and 47% of general dentists had a moderate knowledge of evidence-based endodontic practices, while a high level of knowledge was demonstrated by 35% and 13% of dental practitioners, respectively [73]. A study by Al-Omari et al. found that dentists practicing in North Jordan did not comply with international quality standards [74]. Albahiti et al. found moderately satisfying knowledge of decontamination during RCT, with no differences in this parameter between dentists working in government and private sectors [71].

Apart from the general knowledge score, we also analyzed participants’ answers to particular questions. A majority of the respondents correctly identified EDTA properties and concentrations used in RCT. However, less than a third of the respondents were aware of the reaction occurring between EDTA and sodium hypochlorite, leading to the loss of the antibacterial and tissue-dissolving properties of the latter. None of the participants correctly listed all chelating irrigants used in endodontics. The most recognized endodontic chelating solution was citric acid (80.0%), followed by EDTA (59.8%) and etidronic acid (19.9%). Maleic acid was recognized as an endodontic chelating solution by only 3.7% of the respondents. This may be due to the fact that solutions containing this acid are not currently available in the Russian Federation.

As was mentioned above, the use of CAs is required to dissolve the inorganic components of the smear layer. However, the question of whether to retain or to remove the smear layer remains controversial [10,75]. Pashley hypothesized that a smear layer on the root canal walls can prevent bacterial invasion of the dentinal tubules [76]. In contrast, Williams and Goldman found that the smear layer could not prevent bacterial penetration, but only delayed it [77]. Meryon and Brook did not confirm the effect of the smear layer on the ability of oral bacteria to invade dentine tubules in vitro [78]. On the other hand, a systematic review and meta-analysis by Shahravan et al. concluded that smear layer removal had a significant influence on the effective sealing of the root canal system [10]. A systematic review by Violich and Chandler also advocated the removal of the smear layer for better disinfection of the root canal and better adaptation of filling materials to the canal walls [21]. At the same time, these authors admitted the absence of clinical trials to support this recommendation. According to Virdee et al., a majority of the dental schools in the UK and Ireland taught the irrigation protocol, including the use of both sodium hypochlorite and a chelating agent [79].

In our survey, more than 80% of dental practitioners demonstrated a positive attitude towards the use of CAs in endodontic irrigation. No differences were found between the study subgroups in this parameter. Similarly, the majority of the dentists (81%) in the study by Willershausen indicated that removal of the smear layer played an important role in root canal disinfection [13]. According to our results, more than half of dental practitioners agreed or strongly agreed that root canal irrigation with sodium hypochlorite should be alternated with irrigation using CAs. This approach is recommended in several reports [24,80,81,82].

However, this is not in agreement with the studies showing that EDTA should be used at the end of the shaping instead of alternating EDTA and sodium hypochlorite [18,41].

Also, 45% of the respondents believed that CAs could decrease the effect of sodium hypochlorite and 37% of the respondents believed that CAs could weaken dentin structure. Despite their belief that EDTA has deleterious effects, almost a third of the respondents always used chelating solutions in endodontic irrigation, while only 17% of the respondents did not use chelating solutions at all. In a study by Tošić et al., EDTA was the least-applied irrigant in the two surveys conducted in Serbia [83]. In some of the earlier studies, dental practitioners did not report the use of chelating solutions in their irrigation protocols [84,85].

Several surveys have assessed the removal of the smear layer and 20% to 93% of dental practitioners answered that they aimed to remove it [13,15,16,67,68,86,87]. However, some respondents indicated that they removed the smear layer, using paste- or gel-type chelating agents [14,86,87,88]. According to the literature, paste- or gel-based CAs possess only a lubricating effect and do not remove the smear layer effectively when compared to liquid EDTA [21]. Therefore, the use of these CAs does not result in smear layer removal [88]. In our questionnaire, we avoided the question about smear layer removal and asked the participants about preferred forms of CAs (gel or solution) and the type of chelating solutions used. Our survey revealed that dentists working in the private sector used chelating irrigating solutions more frequently and preferred liquid forms of EDTA to gel-type forms. Regarding the specialties, endodontists used EDTA solution more frequently (87.2%) than general dentists (71.4), although the differences were not significant (*p* = 0.06661). Similarly, in a study by De Grigorio et al., endodontists mainly used EDTA or citric acid solutions (62.2%), while 44.5% of general dentists used EDTA gel and only 24.4% used EDTA solution [87]. Moss et al. reported that EDTA solution was used by 76.1% of endodontists, while gel-type EDTA was used by 15.2% [88]. According to our findings, EDTA was the most commonly used chelating solution, followed by citric acid (irrespective of the diagnosis). This finding is in agreement with those of other surveys, stating that EDTA and citric acid were the most widely used chelating irrigating solutions in endodontics [13,86,87,88].

Most previous studies have reported that the diagnosis could influence the clinician’s choice of the irrigation protocol [13,15,67], but these protocols mainly differed in the use of sodium hypochlorite. In contrast, Vasundhara et al. found that a majority of the respondents did not change their choice of irrigants depending on pulpal and periapical diagnosis [89]. Currently, there is no evidence concerning the benefits of any chelating solution in specific clinical situations. However, we found some differences in the choice of chelating solutions by the survey respondents depending on the diagnosis and clinical case (RCT or reRCT). A slightly greater proportion of dentists preferred using EDTA and citric acid when treating teeth with necrotic pulps (apical periodontitis), but not when treating teeth with vital pulps (pulpitis). This is in agreement with the findings of Moss et al., who reported that some dental schools recommended the combined use of EDTA and sodium hypochlorite specifically in nonvital cases [88]. Interestingly, some dental practitioners (1.9%) reported the use of etidronic acid in reRCT, but not in cases of RCT.

We found a significant correlation between the use of CAs (practice) and the attitude towards CAs and between the use of CAs and the knowledge of CAs. These results are in agreement with Unal et al., who concluded that dentists mainly used techniques, products, and materials currently favored by expert opinion, which influenced their attitudes [90].

We readily acknowledge several limitations to our survey. First, it included dental practitioners from one city, so the results cannot be generalized to the whole country. Second, convenience sampling was used, which is why the survey population was not balanced and included mostly general dentists and dentists working in private clinics. Also, the use of self-reporting data could lead to response bias and affect the accuracy of the findings.

## 5. Conclusions

Dental practitioners showed fair knowledge of the chelating agents used in root canal treatment. Endodontists and dentists employed in private clinics demonstrated significantly better knowledge compared with general dentists and dentists employed in government-funded clinics. The attitude of dental practitioners towards chelating agents was positive irrespective of the specialty and workplace. No differences were found in practice between endodontists and general practitioners; however, the use of chelating agents differed significantly between dentists working in private and government-funded clinics. Practice significantly correlated with attitude towards chelating agents and with knowledge of the topic.

## Figures and Tables

**Table 1:** Participants’ demographic characteristics.

Sociodemographic Data	Count, *n*	Percentage, %
Gender		
Male	115	30.6
Female	261	69.4
Specialty		
Endodontist	47	12.5
General practitioner	329	87.5
Years of clinical experience		
<5	160	42.6
5–10	97	25.8
11–20	75	19.9
>20	44	14.9
Clinic		
Private	85	22.6
Government-funded	291	77.4

**Table 2:** Respondents’ knowledge of chelating agents in endodontic treatment among dental practitioners.

Knowledge	Total	Specialty		Clinic	
		Endodontists	General Practitioners	Private	Government-Funded
Score, points					
Mean (sd)	13.5 (2.4)	14.3 (1.7)	13.4 (2.1)	13.7 (2.0)	13.0 (2.2)
Median (Q1; Q3)	14 (12; 15)	15 (13; 15)	14 (12; 15)	13 (12; 15)	14 (13; 15)
Min, Max	8, 18	10, 28	8, 17	8, 18	8, 17
*p*-value ^1^		0.01185		0.01742	
Level, *n* (%)					
Poor	62 (16.5)	2 (4.3)	60 (18.2)	42 (14.4)	20 (23.5)
Fair	314 (83.5)	45 (95.7)	269 (81.8)	249 (85.6)	65 (76.5)
Good	-	-	-	-	-
*p*-value ^2^		0.01157		0.06625	

^1^ according to Mann–Whitney U test; ^2^ according to Fisher’s exact test.

**Table 3:** Distribution of responses to knowledge questions about chelating agents in endodontic treatment among dental practitioners, *n* (%).

Question	Total	Specialty		Clinic	
		Endodontists	General Practitioners	Private	Government-Funded
EDTA properties					
Correct	313 (83.2)	44 (93.6)	269 (81.8)	248 (85.2)	65 (76.5)
Incorrect	63 (16.8)	3 (6.4)	60 (18.2)	43 (14.8)	20 (23.5)
*p*-value ^1^		0.05744		0.06907	
EDTA concentration					
Correct	295 (78.5)	44 (93.6)	251 (76.4)	235 (80.8)	60 (70.6)
Incorrect	81 (21.5)	3 (6.4)	78 (23.6)	56 (19.2)	25 (29.4)
*p*-value ^1^		0.004399		0.05161	
Chelating solutions used for root canal irrigation					
Correct ^2^	34 (9.0)	7 (14.9)	27 (8.3)	30 (10.3)	4 (4.7)
Incorrect	342 (81.0)	40 (85.1)	302 (91.8)	261 (89.7)	81 (95.3)
*p*-value ^1^		0.1683		0.1346	
Interaction between EDTA and NaOCl					
Correct	87 (23.1)	14 (29.8)	73 (22.2)	71 (24.4)	16 (18.8)
Incorrect	289 (76.9)	33 (70.2)	256 (77.8)	220 (75.6)	69 (81.2)
*p*-value ^1^		0.2682		0.3096	
Interaction between EDTA and CHX					
Correct	20 (5.3)	4 (8.5)	16 (4.9)	14 (4.8)	6 (7.1)
Incorrect	309 (94.7)	43 (91.5)	313 (95.1)	277 (95.2)	79 (92.9)
*p*-value ^1^		0.295		0.4148	

^1^ according to Fisher’s exact test; ^2^ respondents chose at least 3 chelating agents out of 4 correctly; EDTA—ethylenediaminetetraacetic acid; NaOCl—sodium hypochlorite; CHX—chlorhexidine.

**Table 4:** Respondents’ attitude toward chelating agents in endodontic treatment.

Attitude	Total	Specialty		Clinic	
		Endodontists	General Practitioners	Private	Government-Funded
Score					
Mean (sd)	12.8 (2.6)	13.1 (2.8)	12.8 (2.5)	12.8 (2.5)	13.0 (2.8)
Median (Q1; Q3)	13 (11; 15)	12 (11.5; 15)	13 (11; 14)	12 (11; 15)	13 (11; 15)
Min, Max	5, 20	8, 20	5, 20	6, 20	5, 20
*p*-value ^1^		W = 8139, *p*-value = 0.5555		W = 12980, *p*-value = 0.4837	
Level, *n* (%)					
Negative	64 (17.0)	10 (21.3)	54 (16.4)	50 (17.2)	14 (16.5)
Positive	312 (83.0)	37 (78.7)	275 (83.6)	241 (82.8)	71 (83.5)
*p*-value ^2^		0.4089		1.0	

^1^ according to Mann–Whitney U test; ^2^ according to Fisher’s exact test.

**Table 5:** Distribution of responses to attitude questions about chelating agents in endodontic treatment among dental practitioners, *n* (%).

Item	Respondents’ Answers *n* (%)				
	SA	A	N	D	SD
NaOCl should be combined with CAs	125 (33.2)	86 (22.9)	84 (22.3)	49 (13.1)	32 (8.5)
CAs weaken dentin structure	56 (14.9)	83 (22.1)	98 (26.1)	72 (19.1)	67 (17.8)
CAs should be used only in sclerotized root canals	40 (10.6)	60 (16.0)	66 (17.6)	82 (21.8)	128 (34.0)
CAs decrease the effect of NaOCl	94 (25.0)	76 (20.2)	104 (27.6)	42 (11.2)	60 (16.0)

CAs—chelating agents; NaOCl—sodium hypochlorite; SA—strongly agree; A—agree; N—neutral; D—disagree; SD—strongly disagree.

**Table 6:** Dental practitioners’ practice scores.

Practice	Total	Specialty		Clinic	
		Endodontists	General Practitioners	Private	Government-Funded
Mean (sd)	5.1 (1.7)	4.8 (1.2)	4.3 (1.5)	4.5 (1.4)	4.0 (1.7)
Median (Q1; Q3)	5 (5; 6)	5 (4; 6)	4 (4; 5)	6 (4; 8)	4 (3; 5)
Min, Max	0, 8	0, 6	0, 8	0, 8	0, 6
*p*-value ^1^		0.06536		0.1104	

^1^ according to Mann–Whitney U test.

## Data Availability

The datasets used and/or analyzed during the current study are available from the corresponding author upon request.
